# 7-[4-(4-Fluoro­phen­yl)-2-methyl­sulfanyl-1*H*-imidazol-5-yl]tetra­zolo[1,5-*a*]pyridine

**DOI:** 10.1107/S1600536810002680

**Published:** 2010-01-27

**Authors:** Roland Selig, Dieter Schollmeyer, Joachim Schlosser, Wolfgang Albrecht, Stefan Laufer

**Affiliations:** a Eberhard-Karls-University Tübingen, Auf der Morgenstelle 8, 72076 Tübingen, Germany; b University Mainz, Duesbergweg 10-14, 55099 Mainz, Germany; c c-a-i-r biosciences GmbH, Paul-Ehrlich-Strasse 15, 72076 Tübingen, Germany

## Abstract

The crystal structure of the title compound, C_15_H_11_FN_6_S, forms a three-dimensional network stabilized by π–π inter­actions between the imidazole core and the tetra­zole ring of the tetra­zolopyridine­unit; the centroid–centroid distance is 3.627 (1) Å. The crystal structure also displays bifurcated N—H⋯(N,N) hydrogen bonding and C—H⋯F inter­actions. The former involve the NH H atom of the imidazole core and the tetra­zolopyridine N atoms, while the latter involve a methyl H atom, of the methyl­sulfanyl group, and the 4-fluoro­phenyl F atom. In the mol­ecule, the imidazole ring makes dihedral angles of 40.45 (9) and 17.09 (8)°, respectively, with the 4-fluoro­phenyl ring and the tetra­zolopyridine ring mean plane.

## Related literature

For the biological relevance and the development of p38 MAP kinase inhibitors, see: see: Peifer *et al.* (2006 ▶). For the preparation of 2-fluoro-4-[4-(4-fluoro­phen­yl)-2-(methyl­thio)-1*H*-imidazol-5-yl]pyridine, see: Laufer & Liedtke (2006 ▶). For the preparation of tetra­zolopyridines, see: Capelli *et al.* (2008 ▶). 
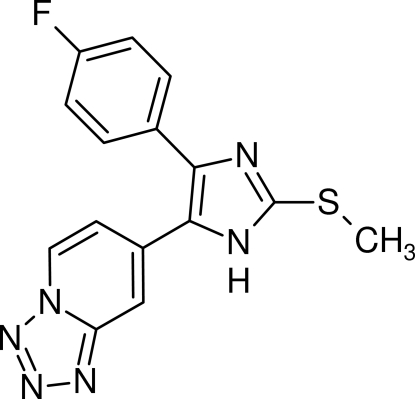


## Experimental

### 

#### Crystal data

C_15_H_11_FN_6_S
*M* *_r_* = 326.36
Monoclinic, 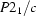

*a* = 9.8342 (7) Å
*b* = 18.1908 (6) Å
*c* = 8.2374 (7) Å
β = 100.292 (3)°
*V* = 1449.89 (17) Å^3^
*Z* = 4
Cu *K*α radiation
μ = 2.17 mm^−1^
*T* = 193 K
0.30 × 0.20 × 0.10 mm

#### Data collection

Enraf–Nonius CAD-4 diffractometer
Absorption correction: ψ scan (*CORINC*; Dräger & Gattow, 1971 ▶) *T*_min_ = 0.866, *T*_max_ = 0.999
5760 measured reflections
2733 independent reflections
2403 reflections with *I* > 2σ(*I*)
*R*_int_ = 0.056
3 standard reflections every 60 min  intensity decay: 3%

#### Refinement

*R*[*F* ^2^ > 2σ(*F* ^2^)] = 0.038
*wR*(*F* ^2^) = 0.107
*S* = 1.06
2733 reflections
210 parameters
H-atom parameters constrained
Δρ_max_ = 0.37 e Å^−3^
Δρ_min_ = −0.30 e Å^−3^

### 

Data collection: *CAD-4 Software* (Enraf–Nonius, 1989 ▶); cell refinement: *CAD-4 Software*; data reduction: *CORINC* (Dräger & Gattow, 1971 ▶); program(s) used to solve structure: *SIR97* (Altomare *et al.*, 1999 ▶); program(s) used to refine structure: *SHELXL97* (Sheldrick, 2008 ▶); molecular graphics: *PLATON* (Spek, 2009 ▶); software used to prepare material for publication: *PLATON*.

## Supplementary Material

Crystal structure: contains datablocks I, global. DOI: 10.1107/S1600536810002680/su2158sup1.cif

Structure factors: contains datablocks I. DOI: 10.1107/S1600536810002680/su2158Isup2.hkl

Additional supplementary materials:  crystallographic information; 3D view; checkCIF report

## Figures and Tables

**Table 1:** Hydrogen-bond geometry (Å, °)

*D*—H⋯*A*	*D*—H	H⋯*A*	*D*⋯*A*	*D*—H⋯*A*
N10—H10⋯N1^i^	0.92	2.06	2.9703 (19)	174
N10—H10⋯N2^i^	0.92	2.60	3.423 (2)	151
C21—H21*B*⋯F19^ii^	0.98	2.44	3.286 (3)	144

Symmetry codes: (i) 
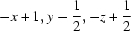
; (ii) 
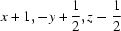
.

**Fig. 1.**
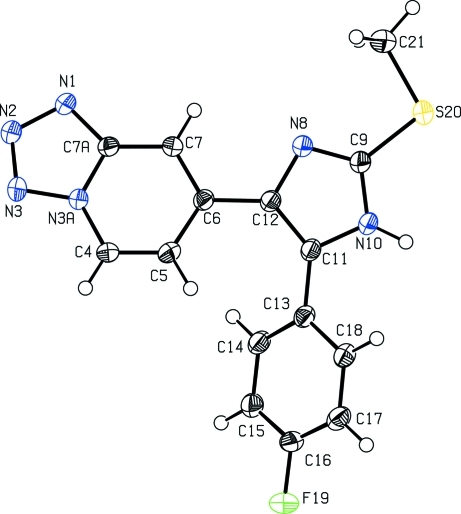
A view of the molecular structure of the title compound. the displacement ellipsoids are drawn at the 50% probability level.

## supplementary crystallographic information

### Comment

Pyridylimidazoles like SB203580 are well known p38 MAP kinase inhibitors (Peifer *et al.*, 2006). The function of the pyridine moiety is to accept a hydrogen bond from the backbone of Met109 in the Hinge region. In the course of our studies we have tried to modify this acceptor system by using the title tetrazolopyridine (Capelli *et al., *2008).

The molecular structure of the title compound is given in Fig. 1, and the geometrical parameters are available in the Supplementary information and the archived CIF. The imidazole ring mean plane makes dihedral angles of 40.45 (9)° and 17.09 (8)° with the 4-fluorophenyl ring and the tetrazolopyridine ring mean plane, respectively.

The crystal structure displays asymmetric bifurcated N—H···N hydrogen bonds involving the tetrazolopyridine N-atoms and the NH H-atom of the the imidazole core (Table 1). There is also a C-H···F interaction involving the methylsulfanyl group and the 4-fluorophenyl F-atom (Table 1). The crystal structure of the title compound forms a three dimensional network stabilized by π-π interactions between the imidazole core and the tetrazole moiety of the tetrazolopyridine group; the centroid···centroid distance is 3.627 (1) Å (Table 1).

### Experimental

A mixture of 300 mg 2-fluoro-4-[4-(4-fluorophenyl)-2-(methylthio)-1*H*-imidazol-5- yl]pyridine (Laufer & Liedtke, 2006) in anhydrous DMF with 100 mg sodium azide was heated at 353 K for 12 h. The solvent was then removed under reduced pressure and the residue was diluted with ethylacetate. The organic phase was washed with water and concentrated under reduced pressure. The residue was purified by flash chromatography with ethyl acetate/hexane (1/1) to yield 153 mg (47%) of the title compound. Crystals suitable for X-ray analysis were obtained by crystallization from methanol.

### Refinement

The H atom attached to N10 was located in a difference Fourier map and refined with a distance restraint of 0.92 (2) Å and U_iso_(H) = 1.2U_eq_(N). The C-bound H-atoms were placed in calculated positions and refined in the riding-model approximation: C-H = 0.95 - 0.98 Å with U_iso_(H) = k × U_eq_(C), where k = 1.2 for H-aromatic and 1.5 for H-methyl.

### Crystal data

**Table:**

C_15_H_11_FN_6_S	*F*(000) = 672
*M**_r_* = 326.36	*D*_x_ = 1.495 Mg m^−^^3^
Monoclinic, *P*2_1_/*c*	Cu *K*α radiation, λ = 1.54178 Å
Hall symbol: -P 2ybc	Cell parameters from 25 reflections
*a* = 9.8342 (7) Å	θ = 65–70°
*b* = 18.1908 (6) Å	µ = 2.17 mm^−^^1^
*c* = 8.2374 (7) Å	*T* = 193 K
β = 100.292 (3)°	Plate, colourless
*V* = 1449.89 (17) Å^3^	0.30 × 0.20 × 0.10 mm
*Z* = 4	

### Data collection

**Table:**

Enraf–Nonius CAD-4 diffractometer	2403 reflections with *I* > 2σ(*I*)
Radiation source: rotating anode	*R*_int_ = 0.056
graphite	θ_max_ = 69.9°, θ_min_ = 4.6°
ω/2θ scans	*h* = −11→11
Absorption correction: ψ scan (CORINC; Dräger & Gattow, 1971)	*k* = −22→22
*T*_min_ = 0.866, *T*_max_ = 0.999	*l* = −10→0
5760 measured reflections	3 standard reflections every 60 min
2733 independent reflections	intensity decay: 3%

### Refinement

**Table:**

Refinement on *F*^2^	Secondary atom site location: difference Fourier map
Least-squares matrix: full	Hydrogen site location: inferred from neighbouring sites
*R*[*F*^2^ > 2σ(*F*^2^)] = 0.038	H-atom parameters constrained
*wR*(*F*^2^) = 0.107	*w* = 1/[σ^2^(*F*_o_^2^) + (0.051*P*)^2^ + 0.5315*P*] where *P* = (*F*_o_^2^ + 2*F*_c_^2^)/3
*S* = 1.06	(Δ/σ)_max_ = 0.001
2733 reflections	Δρ_max_ = 0.37 e Å^−^^3^
210 parameters	Δρ_min_ = −0.30 e Å^−^^3^
0 restraints	Extinction correction: *SHELXL97* (Sheldrick, 2008), Fc^*^=kFc[1+0.001xFc^2^λ^3^/sin(2θ)]^-1/4^
Primary atom site location: structure-invariant direct methods	Extinction coefficient: 0.0014 (2)

### Special details

**Table:**

Geometry. Bond distances, angles etc. have been calculated using the rounded fractional coordinates. All su's are estimated from the variances of the (full) variance-covariance matrix. The cell esds are taken into account in the estimation of distances, angles and torsion angles
Refinement. Refinement of *F*^2^ against ALL reflections. The weighted *R*- factor *wR* and goodness of fit *S* are based on *F*^2^, conventional *R*-factors *R* are based on *F*, with *F* set to zero for negative *F*^2^. The threshold expression of *F*^2^ > σ(*F*^2^) is used only for calculating *R*-factors(gt) *etc*. and is not relevant to the choice of reflections for refinement. *R*-factors based on *F*^2^ are statistically about twice as large as those based on *F*, and *R*- factors based on ALL data will be even larger.

### Fractional atomic coordinates and isotropic or equivalent isotropic displacement parameters (Å^2^)

**Table:**

	*x*	*y*	*z*	*U*_iso_*/*U*_eq_	
S20	0.84423 (5)	0.32567 (2)	0.15709 (8)	0.0423 (2)	
F19	0.09192 (12)	0.08344 (6)	0.37488 (16)	0.0442 (4)	
N1	0.29691 (15)	0.61704 (7)	0.31439 (19)	0.0284 (4)	
N2	0.18289 (15)	0.63452 (8)	0.3762 (2)	0.0315 (4)	
N3	0.12953 (15)	0.57831 (8)	0.4381 (2)	0.0304 (4)	
N3A	0.21213 (13)	0.52053 (7)	0.41594 (17)	0.0232 (4)	
N8	0.62050 (14)	0.39105 (8)	0.25381 (18)	0.0265 (4)	
N10	0.61319 (14)	0.26910 (7)	0.24463 (18)	0.0271 (4)	
C4	0.20076 (17)	0.44913 (9)	0.4666 (2)	0.0267 (5)	
C5	0.29443 (17)	0.40040 (9)	0.4318 (2)	0.0264 (5)	
C6	0.40054 (16)	0.42075 (8)	0.3415 (2)	0.0221 (4)	
C7	0.41195 (16)	0.49369 (9)	0.2991 (2)	0.0231 (4)	
C7A	0.31553 (16)	0.54434 (9)	0.3388 (2)	0.0230 (4)	
C9	0.68541 (17)	0.33087 (9)	0.2239 (2)	0.0275 (5)	
C11	0.49088 (16)	0.29071 (9)	0.2895 (2)	0.0239 (5)	
C12	0.49810 (16)	0.36671 (9)	0.2974 (2)	0.0233 (5)	
C13	0.38534 (16)	0.23580 (9)	0.3073 (2)	0.0241 (5)	
C14	0.24524 (17)	0.24835 (9)	0.2461 (2)	0.0286 (5)	
C15	0.14573 (18)	0.19732 (10)	0.2673 (2)	0.0313 (5)	
C16	0.18916 (19)	0.13307 (9)	0.3491 (2)	0.0301 (5)	
C17	0.32598 (19)	0.11634 (9)	0.4043 (2)	0.0297 (5)	
C18	0.42396 (18)	0.16837 (9)	0.3835 (2)	0.0266 (5)	
C21	0.8749 (2)	0.42202 (11)	0.1351 (3)	0.0420 (7)	
H4	0.12940	0.43470	0.52390	0.0320*	
H5	0.29010	0.35100	0.46820	0.0320*	
H7	0.48390	0.50930	0.24390	0.0280*	
H10	0.64590	0.22270	0.23220	0.0320*	
H14	0.21790	0.29280	0.18890	0.0340*	
H15	0.05050	0.20630	0.22690	0.0380*	
H17	0.35240	0.07040	0.45520	0.0360*	
H18	0.51900	0.15820	0.42160	0.0320*	
H21A	0.80250	0.44270	0.05010	0.0630*	
H21B	0.96520	0.42910	0.10280	0.0630*	
H21C	0.87380	0.44690	0.24030	0.0630*	

### Atomic displacement parameters (Å^2^)

**Table:**

	*U*^11^	*U*^22^	*U*^33^	*U*^12^	*U*^13^	*U*^23^
S20	0.0311 (3)	0.0261 (3)	0.0757 (4)	0.0036 (2)	0.0258 (2)	−0.0011 (2)
F19	0.0400 (6)	0.0307 (6)	0.0661 (8)	−0.0095 (5)	0.0210 (6)	0.0042 (5)
N1	0.0261 (7)	0.0188 (7)	0.0413 (8)	0.0027 (5)	0.0087 (6)	0.0029 (6)
N2	0.0264 (7)	0.0217 (7)	0.0478 (9)	0.0046 (6)	0.0104 (6)	0.0033 (6)
N3	0.0264 (7)	0.0230 (7)	0.0436 (9)	0.0066 (6)	0.0112 (6)	0.0017 (6)
N3A	0.0207 (6)	0.0193 (6)	0.0304 (7)	0.0018 (5)	0.0066 (5)	0.0006 (5)
N8	0.0218 (7)	0.0208 (7)	0.0377 (8)	0.0005 (5)	0.0073 (6)	−0.0022 (6)
N10	0.0239 (7)	0.0182 (6)	0.0395 (8)	0.0026 (5)	0.0067 (6)	−0.0020 (6)
C4	0.0273 (8)	0.0208 (8)	0.0342 (9)	−0.0014 (6)	0.0113 (7)	0.0034 (7)
C5	0.0304 (8)	0.0185 (8)	0.0311 (9)	−0.0003 (6)	0.0076 (7)	0.0027 (7)
C6	0.0209 (7)	0.0192 (7)	0.0255 (8)	0.0000 (6)	0.0019 (6)	−0.0016 (6)
C7	0.0202 (7)	0.0204 (7)	0.0293 (8)	−0.0014 (6)	0.0059 (6)	−0.0012 (6)
C7A	0.0211 (7)	0.0191 (7)	0.0286 (8)	−0.0018 (6)	0.0037 (6)	0.0000 (6)
C9	0.0219 (8)	0.0219 (8)	0.0390 (9)	0.0005 (6)	0.0062 (7)	−0.0014 (7)
C11	0.0228 (8)	0.0194 (8)	0.0287 (8)	0.0020 (6)	0.0027 (6)	−0.0015 (6)
C12	0.0221 (8)	0.0198 (8)	0.0275 (8)	0.0007 (6)	0.0031 (6)	−0.0004 (6)
C13	0.0265 (8)	0.0193 (8)	0.0262 (8)	0.0003 (6)	0.0043 (6)	−0.0028 (6)
C14	0.0283 (9)	0.0198 (8)	0.0364 (10)	0.0018 (7)	0.0022 (7)	0.0005 (7)
C15	0.0246 (8)	0.0256 (8)	0.0432 (10)	0.0009 (7)	0.0044 (7)	−0.0034 (8)
C16	0.0320 (9)	0.0227 (8)	0.0382 (9)	−0.0057 (7)	0.0133 (7)	−0.0041 (7)
C17	0.0389 (10)	0.0212 (8)	0.0299 (9)	0.0016 (7)	0.0088 (7)	0.0025 (7)
C18	0.0280 (8)	0.0222 (8)	0.0288 (8)	0.0027 (6)	0.0026 (7)	−0.0005 (6)
C21	0.0417 (11)	0.0295 (10)	0.0608 (13)	−0.0047 (8)	0.0255 (10)	−0.0009 (9)

### Geometric parameters (Å, °)

**Table:**

S20—C9	1.7488 (18)	C11—C12	1.385 (2)
S20—C21	1.793 (2)	C11—C13	1.467 (2)
F19—C16	1.359 (2)	C13—C18	1.399 (2)
N1—N2	1.350 (2)	C13—C14	1.399 (2)
N1—C7A	1.345 (2)	C14—C15	1.382 (2)
N2—N3	1.295 (2)	C15—C16	1.378 (2)
N3—N3A	1.361 (2)	C16—C17	1.375 (3)
N3A—C4	1.375 (2)	C17—C18	1.383 (2)
N3A—C7A	1.362 (2)	C4—H4	0.9500
N8—C9	1.313 (2)	C5—H5	0.9500
N8—C12	1.389 (2)	C7—H7	0.9500
N10—C9	1.356 (2)	C14—H14	0.9500
N10—C11	1.377 (2)	C15—H15	0.9500
N10—H10	0.9200	C17—H17	0.9500
C4—C5	1.346 (2)	C18—H18	0.9500
C5—C6	1.434 (2)	C21—H21A	0.9800
C6—C7	1.382 (2)	C21—H21B	0.9800
C6—C12	1.464 (2)	C21—H21C	0.9800
C7—C7A	1.402 (2)		
			
C9—S20—C21	98.90 (9)	C11—C13—C14	121.46 (15)
N2—N1—C7A	105.98 (13)	C11—C13—C18	120.11 (15)
N1—N2—N3	112.64 (14)	C13—C14—C15	121.26 (15)
N2—N3—N3A	105.28 (14)	C14—C15—C16	117.82 (16)
N3—N3A—C4	127.32 (14)	F19—C16—C17	118.39 (15)
N3—N3A—C7A	109.28 (13)	F19—C16—C15	118.32 (16)
C4—N3A—C7A	123.36 (14)	C15—C16—C17	123.28 (17)
C9—N8—C12	104.82 (14)	C16—C17—C18	118.02 (15)
C9—N10—C11	107.42 (13)	C13—C18—C17	121.08 (16)
C11—N10—H10	129.00	N3A—C4—H4	121.00
C9—N10—H10	123.00	C5—C4—H4	121.00
N3A—C4—C5	117.50 (15)	C4—C5—H5	119.00
C4—C5—C6	121.98 (15)	C6—C5—H5	119.00
C5—C6—C7	118.59 (14)	C6—C7—H7	121.00
C5—C6—C12	121.68 (14)	C7A—C7—H7	121.00
C7—C6—C12	119.71 (14)	C13—C14—H14	119.00
C6—C7—C7A	118.90 (15)	C15—C14—H14	119.00
N3A—C7A—C7	119.48 (14)	C14—C15—H15	121.00
N1—C7A—N3A	106.81 (14)	C16—C15—H15	121.00
N1—C7A—C7	133.70 (15)	C16—C17—H17	121.00
S20—C9—N8	126.57 (13)	C18—C17—H17	121.00
S20—C9—N10	120.80 (12)	C13—C18—H18	119.00
N8—C9—N10	112.59 (15)	C17—C18—H18	119.00
N10—C11—C12	104.93 (14)	S20—C21—H21A	109.00
C12—C11—C13	134.93 (15)	S20—C21—H21B	109.00
N10—C11—C13	120.00 (14)	S20—C21—H21C	109.00
N8—C12—C11	110.22 (14)	H21A—C21—H21B	109.00
C6—C12—C11	130.65 (15)	H21A—C21—H21C	109.00
N8—C12—C6	119.13 (14)	H21B—C21—H21C	109.00
C14—C13—C18	118.41 (15)		
			
C21—S20—C9—N8	−3.16 (18)	C5—C6—C12—C11	18.8 (3)
C21—S20—C9—N10	174.43 (15)	C7—C6—C12—N8	16.8 (2)
N2—N1—C7A—C7	178.07 (18)	C7—C6—C12—C11	−162.78 (17)
N2—N1—C7A—N3A	−0.43 (18)	C5—C6—C12—N8	−161.63 (15)
C7A—N1—N2—N3	0.4 (2)	C12—C6—C7—C7A	178.46 (15)
N1—N2—N3—N3A	−0.1 (2)	C6—C7—C7A—N3A	−0.9 (2)
N2—N3—N3A—C7A	−0.15 (18)	C6—C7—C7A—N1	−179.22 (18)
N2—N3—N3A—C4	177.50 (16)	N10—C11—C12—N8	1.62 (18)
N3—N3A—C7A—N1	0.37 (18)	N10—C11—C12—C6	−178.74 (16)
N3—N3A—C4—C5	−179.88 (15)	C13—C11—C12—N8	−173.91 (17)
C7A—N3A—C4—C5	−2.5 (2)	C13—C11—C12—C6	5.7 (3)
C4—N3A—C7A—C7	3.9 (2)	N10—C11—C13—C14	−136.99 (17)
N3—N3A—C7A—C7	−178.39 (15)	N10—C11—C13—C18	41.5 (2)
C4—N3A—C7A—N1	−177.39 (15)	C12—C11—C13—C14	38.0 (3)
C12—N8—C9—S20	177.95 (13)	C12—C11—C13—C18	−143.46 (19)
C9—N8—C12—C11	−1.15 (18)	C11—C13—C14—C15	−178.13 (15)
C12—N8—C9—N10	0.20 (19)	C18—C13—C14—C15	3.3 (2)
C9—N8—C12—C6	179.17 (15)	C11—C13—C18—C17	178.80 (15)
C9—N10—C11—C12	−1.45 (18)	C14—C13—C18—C17	−2.6 (2)
C9—N10—C11—C13	174.90 (15)	C13—C14—C15—C16	−0.8 (2)
C11—N10—C9—S20	−177.08 (12)	C14—C15—C16—F19	178.12 (15)
C11—N10—C9—N8	0.82 (19)	C14—C15—C16—C17	−2.6 (3)
N3A—C4—C5—C6	−1.7 (2)	F19—C16—C17—C18	−177.45 (15)
C4—C5—C6—C7	4.4 (2)	C15—C16—C17—C18	3.3 (3)
C4—C5—C6—C12	−177.10 (16)	C16—C17—C18—C13	−0.6 (2)
C5—C6—C7—C7A	−3.0 (2)		

### Hydrogen-bond geometry (Å, °)

**Table:**

*D*—H···*A*	*D*—H	H···*A*	*D*···*A*	*D*—H···*A*
N10—H10···N1^i^	0.92	2.06	2.9703 (19)	174
N10—H10···N2^i^	0.92	2.60	3.423 (2)	151
C7—H7···N8	0.95	2.53	2.847 (2)	100
C21—H21B···F19^ii^	0.98	2.44	3.286 (3)	144

Symmetry codes: (i) −*x*+1, *y*−1/2, −*z*+1/2; (ii) *x*+1, −*y*+1/2, *z*−1/2.

## References

[bb1] Altomare, A., Burla, M. C., Camalli, M., Cascarano, G. L., Giacovazzo, C., Guagliardi, A., Moliterni, A. G. G., Polidori, G. & Spagna, R. (1999). *J. Appl. Cryst.* **32**, 115–119.

[bb2] Capelli, A., Giuliani, G., Anzini, M., Riitano, D., Giorgi, G. & Vomero, S. (2008). *Bioorg. Med. Chem.* **16**, 6850–6859. 10.1016/j.bmc.2008.05.067 18554914

[bb3] Dräger, M. & Gattow, G. (1971). *Acta Chem. Scand.* **25**, 761–762.

[bb4] Enraf–Nonius (1989). *CAD-4 Software* Enraf–Nonius, Delft, The Netherlands.

[bb5] Laufer, S. & Liedtke, A. (2006). *Tetrahedron Lett.* **47**, 7199–7203.

[bb6] Peifer, C., Wagner, G. & Laufer, S. (2006). *Curr. Top. Med. Chem.* **6**, 113–149. 10.2174/156802606775270323 16454763

[bb7] Sheldrick, G. M. (2008). *Acta Cryst.* A**64**, 112–122. 10.1107/S0108767307043930 18156677

[bb8] Spek, A. L. (2009). *Acta Cryst.* D**65**, 148–155. 10.1107/S090744490804362X PMC2631630 19171970

